# Severe Rectal Hemorrhage in Adolescents: An Unusual Presentation of Meckel’s Diverticulum

**DOI:** 10.7759/cureus.84601

**Published:** 2025-05-22

**Authors:** Bayan Matarneh, Elizabeth Washnock-Schmid, Andrea Bedway

**Affiliations:** 1 Pediatrics and Neonatology, Children’s Hospital of Michigan, Detroit, USA; 2 Surgery, Case Western Reserve University, Cleveland, USA

**Keywords:** child and adolescent, lower gastrointestinal bleeding, meckel’s diverticulum, red blood cell transfusion, tc99m sestamibi scan

## Abstract

Lower gastrointestinal bleeding in children can be a significant cause of distress and can potentially lead to morbidity and mortality. We present the case of a 15-year-old boy who presented with severe rectal bleeding and required multiple blood transfusions. Further investigation and imaging revealed a Meckel’s diverticulum, which is uncommon in this age group, emphasizing the importance of expanding differential diagnosis when confronted with challenging cases.

## Introduction

Lower gastrointestinal (GI) bleeding is defined as any bleeding distal to the ligament of Treitz. It is a very concerning presentation and can lead to significant concerns, especially in the pediatric population. Unlike upper GI bleeding, which typically presents with hematemesis or melena, lower GI bleeding more often presents as hematochezia. When confronted with lower GI bleeding [1], it is important to keep an open mindset and consider a broad set of differential diagnoses, which could include inflammatory bowel disease, milk protein allergy, vascular lesions, anal fissures, and hemorrhoids. The most common causes vary by age: anal fissures and infectious or allergic colitis in infants, Meckel’s diverticulum and polyps in younger children, and Inflammatory bowel disease (IBD) in adolescents. Most of these causes are self-limiting; however, some adolescents may present with life-threatening GI bleeding and require swift intervention. For instance, anal fissures account for up to 90% of rectal bleeding in infants, while Meckel’s diverticulum is the leading cause of significant painless GI bleeding in children under two years of age [2].

It is essential to identify, revitalize, and stabilize patients presenting with life-threatening bleeding while gathering a focused history and performing a thorough examination. Rapid identification and resuscitation of GI bleeding are often life-saving.

After achieving stability, we usually begin GI bleed work-up with simple laboratory testing, including complete blood count, basic metabolic panel, stool studies, inflammatory markers, and coagulation profile. Imaging should also be considered, especially if the patient has a recent surgical history. Abdominal ultrasound and X-ray are fast and effective for identifying free fluid within the peritoneal cavity in an emergent situation. CT and MRI may be considered when patients are stable without an apparent cause of bleeding on initial imaging. Finally, additional specialty services may be considered, as, in this case, pediatric surgery, gastroenterology, and hematology were consulted.

## Case presentation

A 15-year-old previously medically healthy male presented with a two-day history of bloody stool. On the previous evening, he had a watery bowel movement with deep red-purple blood that filled the toilet bowl. He also had mild abdominal pain that resolved with defecation. After the presentation, he proceeded to have painless, watery, and bloody bowel movements and multiple episodes of bloody stools containing large clots. The patient usually had one to two bowel movements a day and had no history of anal fissures or hemorrhoids. Bleeding was witnessed by his caregiver, and the estimated volume was significant enough to prompt urgent medical attention. He had no previous nausea, vomiting, hematuria, or skin bruising. The patient had a recent history of an upper respiratory tract infection but denied receiving antibiotics. There was no history of traveling. The patient denied fever, weight loss, night sweats, chest pain, palpitation, rashes, or known sick contacts. The patient ate a well-balanced diet of primarily home-cooked meals and reported no recent weight loss.

Upon arrival at the emergency department, the patient was hemodynamically stable. The patient continued to have frequent bowel movements with a significant amount of blood. On physical examination, the patient was hemodynamically stable and appeared pale but in no distress. Mild conjunctival and oral mucosal pallor were noted. The abdomen was soft with mild lower quadrant tenderness. Rectal examination showed no fissures or hemorrhoids, but fresh red blood was present. The tone was normal, and no masses were palpated. His initial hemoglobin was 9.2 mg/dL, and repeat hemoglobin levels were down-trending to 7.6, 7.3, and 6.5 mg/dL, dropping to a low of 5.6 mg/dL within five days of hospitalization. He received five units of packed red blood cells, and the gastroenterology team performed an endoscopy and colonoscopy. Imaging revealed bright red blood with advancement into the terminal ileum. Labs, including a comprehensive metabolic panel, C-reactive protein, and erythrocyte sedimentation rate, were normal, except for a mildly low albumin level of 3.6 g/dL. Additional testing, including *Clostridium difficile* Ag and toxins, fecal calprotectin, stool culture, and tuberculosis test (interferon-gamma release assay), was negative. Table 1 presents laboratory data during admission.

**Table 1:** Laboratory findings.

Laboratory value	Reference range	Three days before presentation	On the day of presentation	One day after admission	After urea tablets	After normal saline boluses	One week after discharge
Hemoglobin	13–17 g/dL	10.2	8.9	9	9	9.7	9.4
White blood cells	4–10 k/µL	1.72	1.01	0.97	1.35	2.03	3.71
Platelets	150–300 k/µL	59	58	57	59	64	100
Creatinine	0.7–1.3 mg/dL	0.91	0.87	0.66	0.69	0.71	0.81
Blood urea nitrogen	6–23 mg/dL	15	14	10	27	23	11
Serum osmolality	275–295 mOst/kg	280	255	248	267	270	274
Sodium	136–145 mmol/L	134	121	122	125	129	136
Urine sodium	Variable	–	92	115	55	67	133
Urine osmolality	Variable	–	558	432	559	587	394

## Discussion

Meckel’s diverticulum (MD) is the most common congenital anomaly of the GI tract [3]. It is characterized by a small pouch in the wall of the intestine and results from incomplete obliteration of the omphalomesenteric duct during fetal development [4]. It typically arises from the antimesenteric border of the ileum, approximately 60 cm (2 feet) proximal to the ileocecal valve. Meckel’s diverticulum is a true diverticulum, meaning it contains all layers of the intestinal wall. The remnant of the omphalomesenteric duct may also be connected to the umbilicus via a fibrous band, which can lead to volvulus or obstruction in rare cases [5]. Common teaching describes using the rule of 2 when discussing Meckel’s diverticulum, which is commonly known as a prevalence rate of 2%, a male-to-female ratio of 2:1, an incidence rate of 2% for symptomatic Meckel’s diverticulum, the presence of symptoms before the age of two years, a location at a distance of 2 feet to the ileocecal valve, a diverticular length of 2 inches, and two types of ectopic tissues [6].

Most individuals with Meckel’s diverticulum remain asymptomatic throughout their lives. However, complications can arise, including bleeding, obstruction, and inflammation. Bleeding most commonly occurs due to the presence of ectopic gastric mucosa within the diverticulum, which secretes acid that causes ulceration of the adjacent ileal mucosa. This results in painless, often profuse hematochezia. Less commonly, ectopic pancreatic tissue or mucosal irritation from retained food can also lead to inflammation and bleeding [7].

The patient in our case continued to have gross bleeding. An endoscopy and colonoscopy were performed by the gastroenterology team, which revealed bright red blood with advancement into the terminal ileum. Additionally, these studies ruled out other causes for recurrent bleeding, such as hemorrhoids, gastric ulcers or polyps, or bowel disease, and, in such cases, offer immediate surgical intervention.

**Figure 1:**
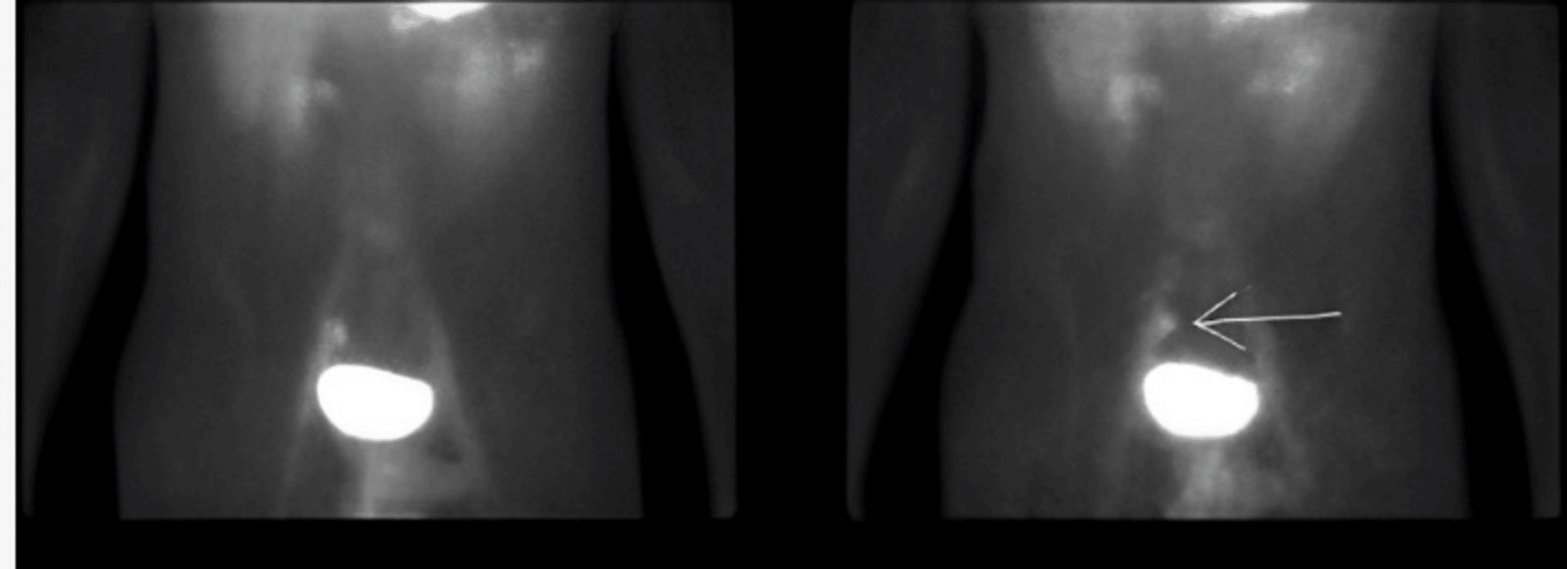
Nuclear medicine Meckel scan using technetium 99m (Tc99m) pertechnetate. Focal accumulation of Tc99m in the right lower abdomen reflecting the uptake in the ectopic gastric mucosa in a Meckel’s diverticulum.

Additional workup in this patient constituted a 99mTc scan, revealing suspicion of Meckel’s diverticulum (Figure 1). After consulting pediatric surgery, an exploratory laparotomy was performed, with resection and later pathological confirmation of Meckel’s diverticulum. Similar presentations have been described in the literature, with studies noting that adolescents with Meckel’s diverticulum may present with significant lower GI bleeding despite lacking the typical age or symptom profile. In some reports, diagnosis was made only after inconclusive endoscopic evaluations, with surgical exploration or nuclear imaging ultimately revealing the diverticulum. These findings support the notion that Meckel’s diverticulum should remain on the differential even in older children, particularly when bleeding is brisk, painless, and unexplained by more common etiologies [8].

While these guidelines are helpful in diagnosis, some cases may not meet these criteria. In this case, irregular presentations are critical to help broaden the differential diagnosis, leading to expedient diagnosis and reduced mortality. The patient described in this case was beyond the expected age of presentation for Meckel’s diverticulum and experienced an acute onset of painless rectal bleeding, requiring multiple blood transfusions. While the diagnosis of Meckel’s diverticulum was made, it was not high on our initial differential, leading to delayed treatment. While our patient recovered without complications, this may not be the case for all. Missed or delayed diagnosis of Meckel’s diverticulum, especially in older children, can lead to ongoing bleeding, hemodynamic instability, and unnecessary workup. This case highlights the importance of maintaining Meckel’s diverticulum in the differential diagnosis for painless lower GI bleeding, regardless of age, and using appropriate imaging early in the course of evaluation. Meckel’s diverticulum can mimic several conditions depending on its presentation. In cases of painless lower GI bleeding, differentials include juvenile polyps, intussusception, and IBD. When presenting with appendicitis, appendicitis itself, mesenteric adenitis, or ovarian torsion should be considered. Bowel obstruction may suggest volvulus or adhesions [9]. By broadening our differential in older adolescents with painless bleeding, monitoring hemoglobin, and consulting appropriately, we can diagnose and treat Meckel’s diverticulum more effectively.

## Conclusions

Meckel’s diverticulum should be considered in the differential diagnosis of painless lower GI bleeding, even in adolescents. Special attention should be given to evaluating patients, as the bleeding from Meckel’s diverticulum can be severe and require multiple blood transfusions. Early diagnosis could be life-saving.
